# Supplementary figures and images for: Deciphering the role of the lncRNA TRIBAL in hepatocyte models
Source: PLoS One. 2025 Sep 2;20(9):e0322975. doi: 10.1371/journal.pone.0322975 (PMC12404505; doi:10.1371/journal.pone.0322975)

Fig 3A

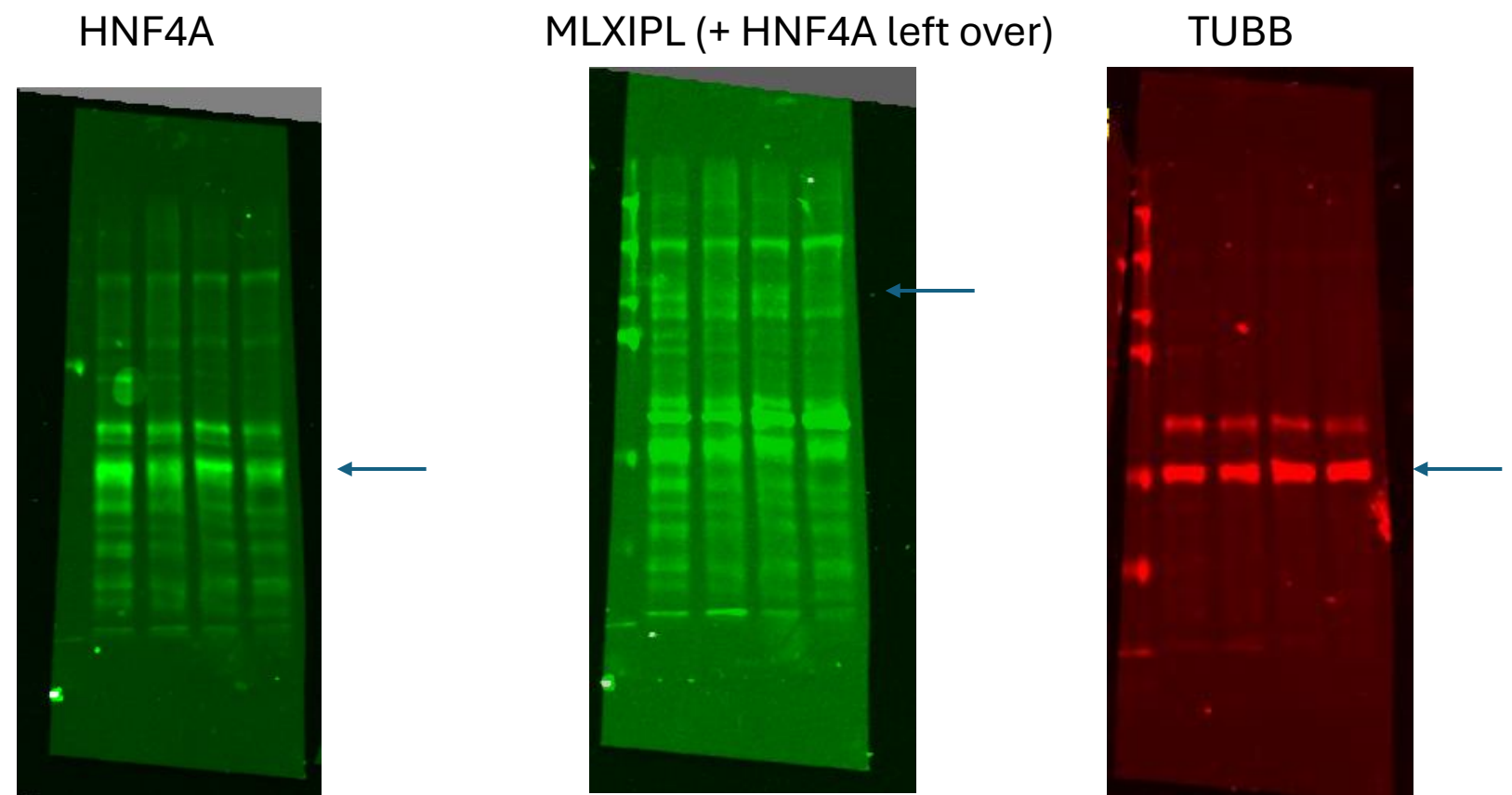

Fig 3B

MLXIPL

HNF4A (+ MLXIPL left over)

TUBB

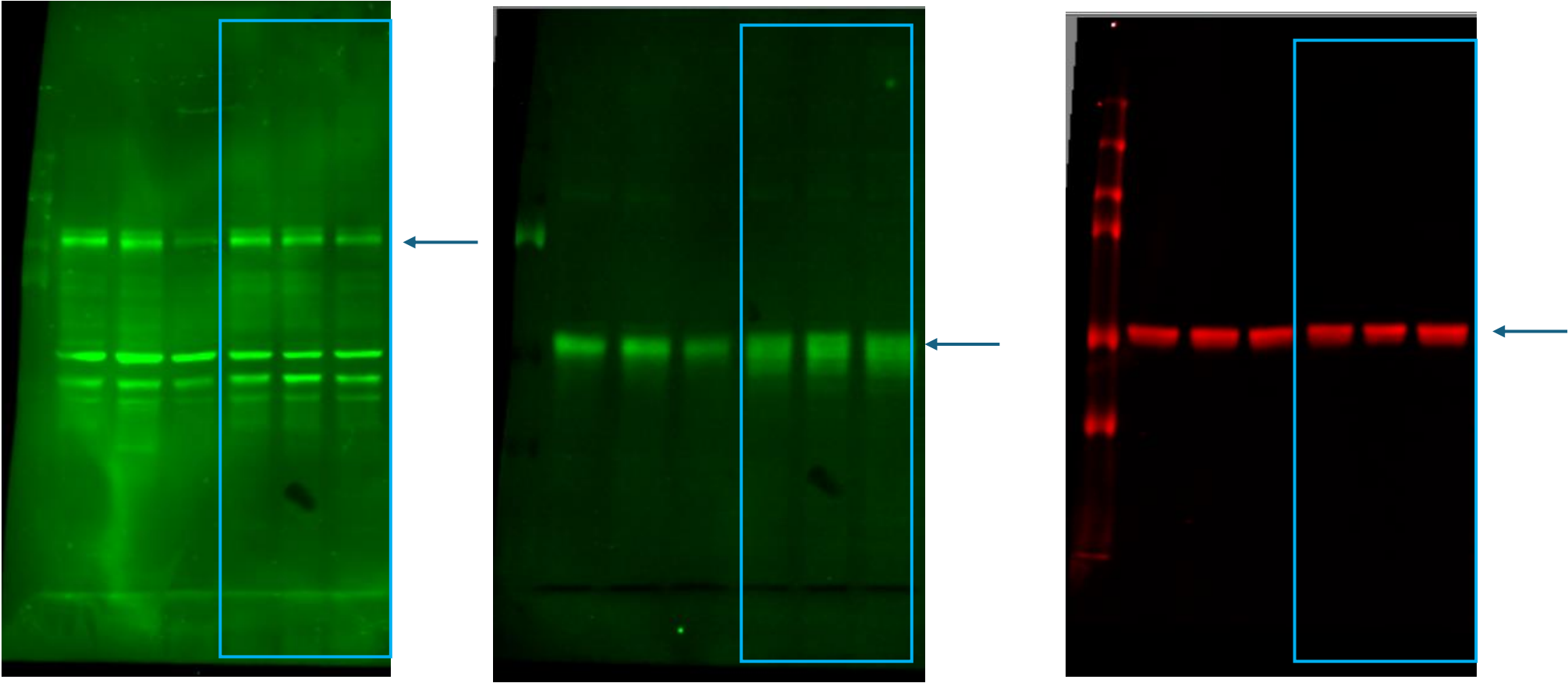

Supplement: S1 File — (PDF) [file pone.0322975.s001.pdf]

S1 Fig.

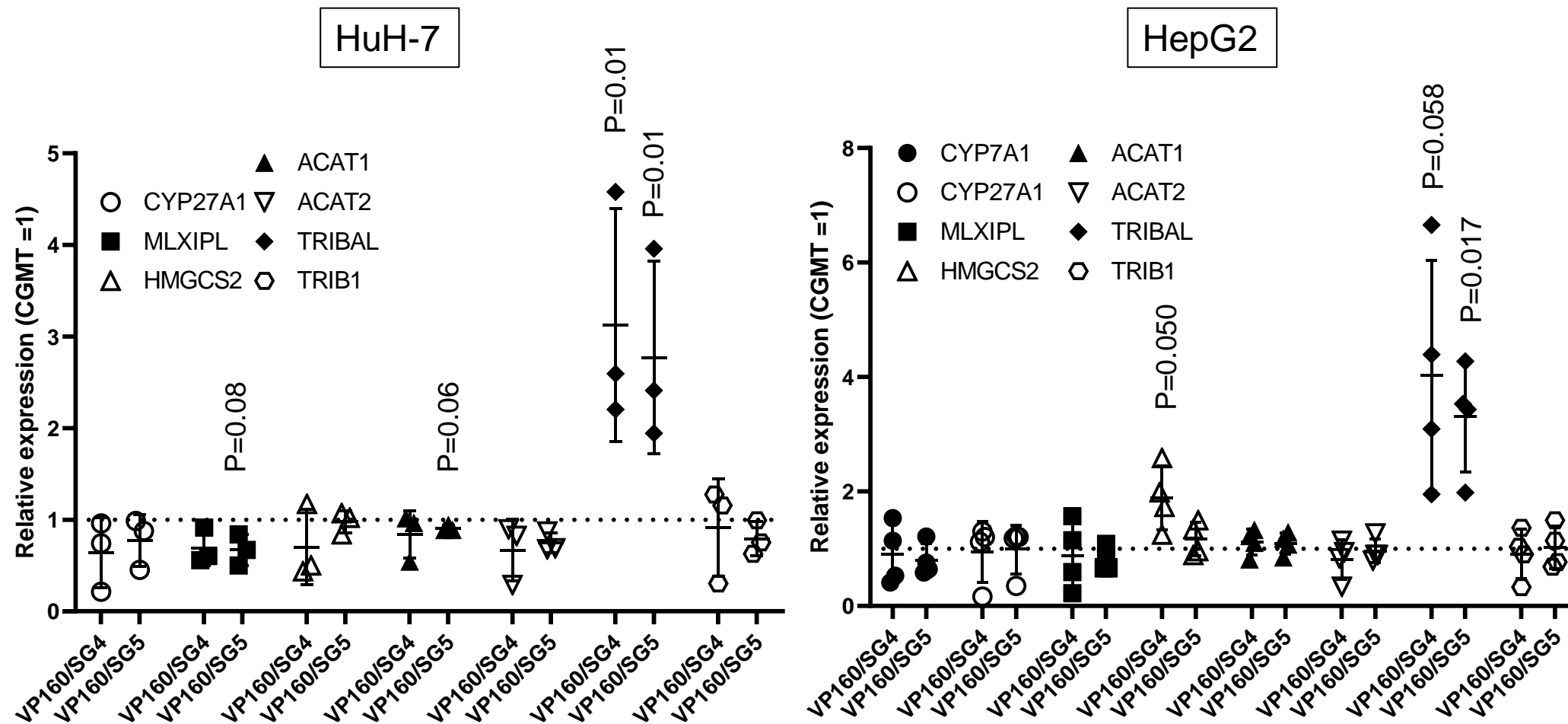

Supplement: S1 Fig — Cells were transfected for 72 h with a plasmid (dCAS9-VP160) encoding the dCAS9-CRISPRa construct and sgRNA sequences (sg4 or sg5) targeting the promoter region of TRIBAL. Each point represents a biological replicate. Statistical significance was assessed using a one-sample t-test using a theoretical control value of 1 in Prism. Nominal P values approaching (p ≤ to 0.1) or surpassing nominal significance are shown. CYP7A1 abundance in HuH-7 cells was too low to be reproducibly measured. (PDF) [file pone.0322975.s003.pdf]

S2 Fig.

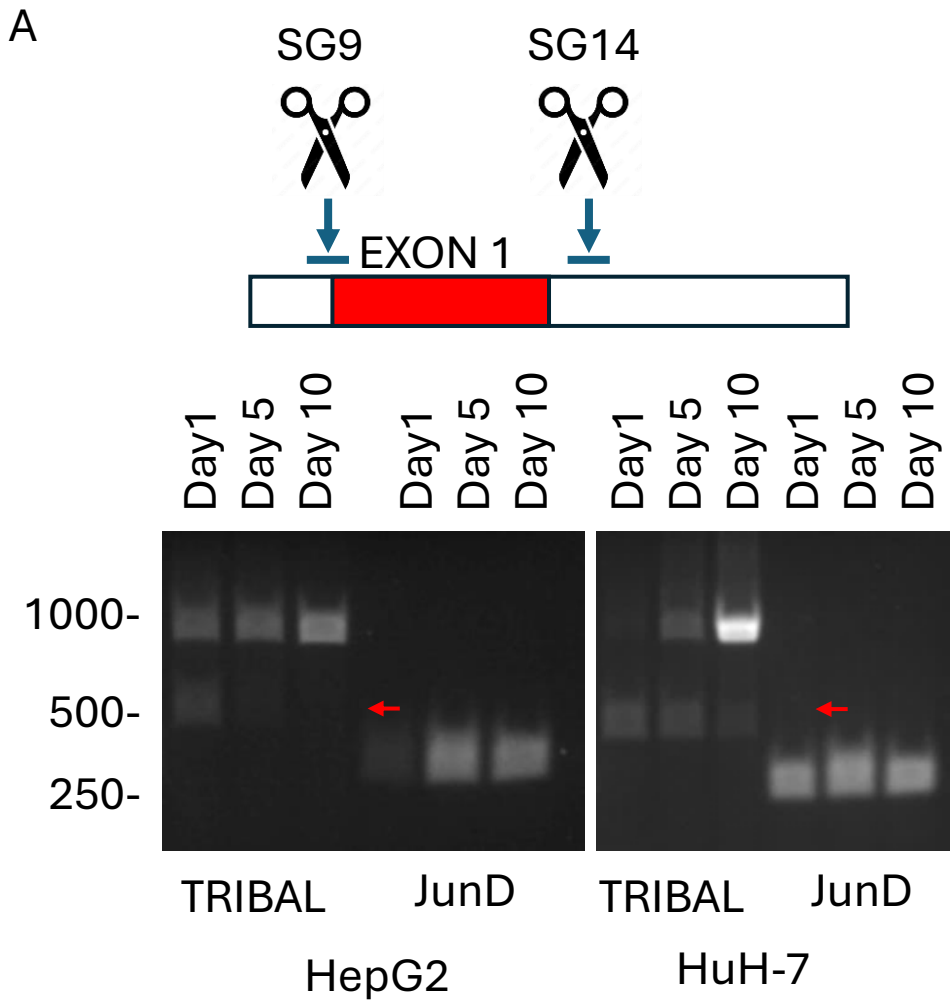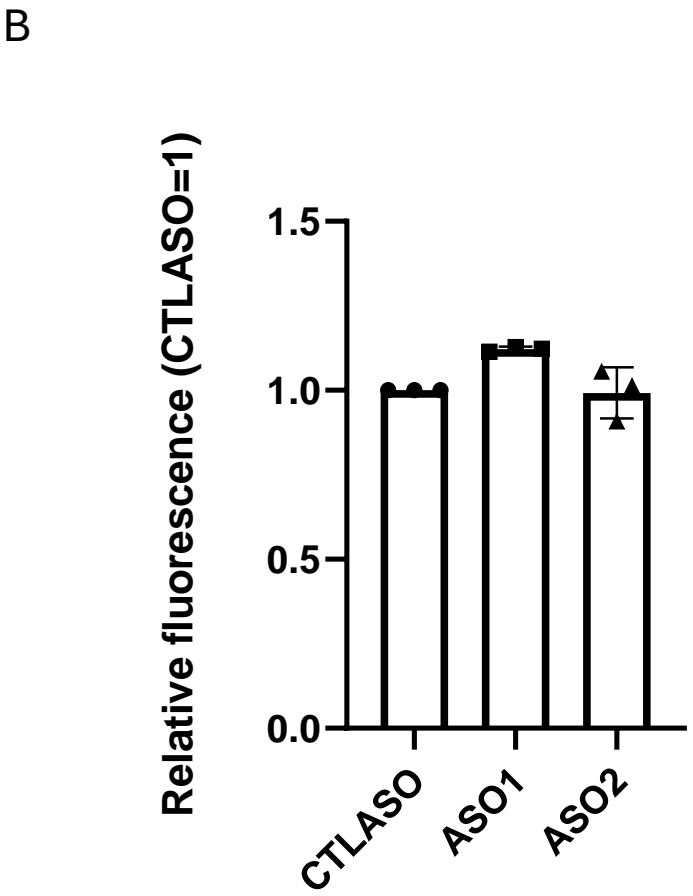

Supplement: S2 Fig — A, TRIBAL was targeted with CRISPR and 2 single guide RNAs flanking exon 1 (sg9 and sg14). The schema of sgRNA positions is shown at the top. The red arrow points to the position of the deleted allele. The experiment was repeated thrice (HepG2) or twice (HuH-7), with similar results. DNA from whole cell lysates was amplified with primers flanking the TRIBAL sgRNA cognate sites or targeting JunD (positive control). B, Impact of TRIBAL ASOs on HepG2 cell proliferation. A one-tailed t-test, performed under the hypothesis that TRIBAL suppression should reduce cell proliferation, showed no significant difference. (PDF) [file pone.0322975.s004.pdf]

S3 Fig.

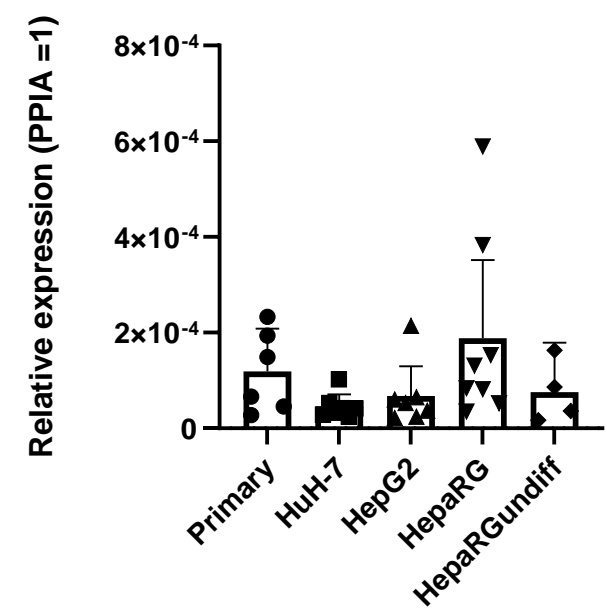

Supplement: S3 Fig — TRIBAL expression was measured in cell models treated with the CTLASO for 72 h. Each point represents a distinct biological replicate. Expression is expressed relative to PPIA. To simplify visualization, error bars below the mean are not shown. (PDF) [file pone.0322975.s005.pdf]

S4 Fig.

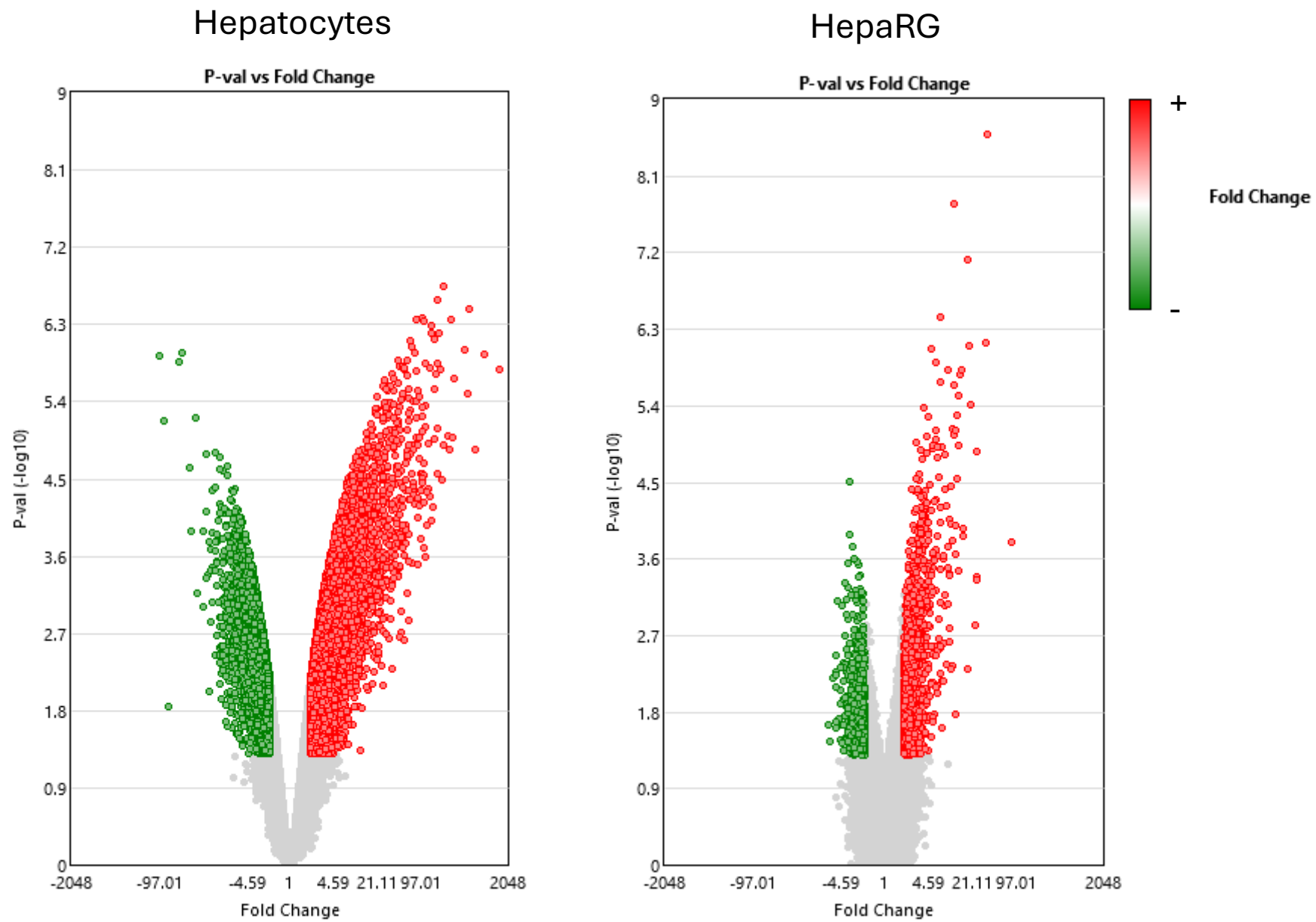

Supplement: S4 Fig — Volcano plots of array data from hepatocytes and HepaRG cells treated with TRIBAL ASO2 (vs CTLASO). Nominal hits experiencing at least 2-fold absolute change in expression are colored. (PDF) [file pone.0322975.s006.pdf]

**S5 Fig.**

**A**

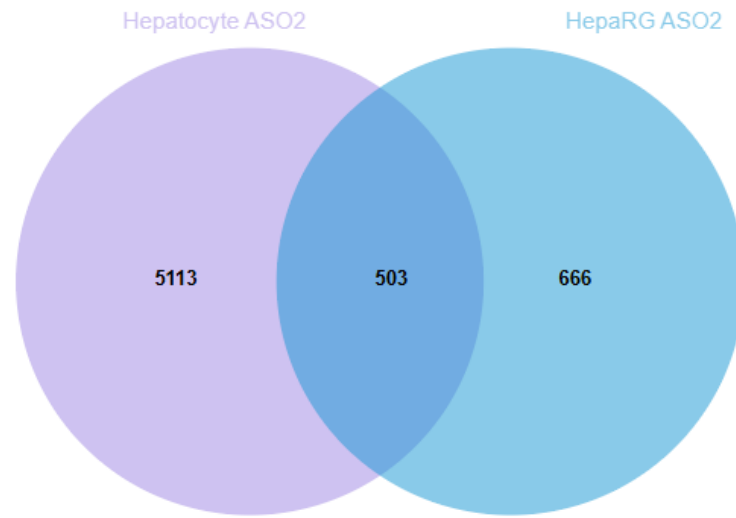

**B**

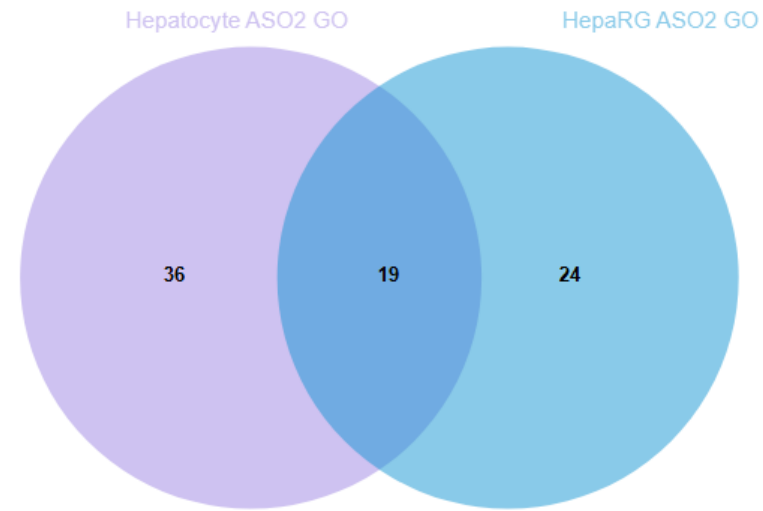

**C**

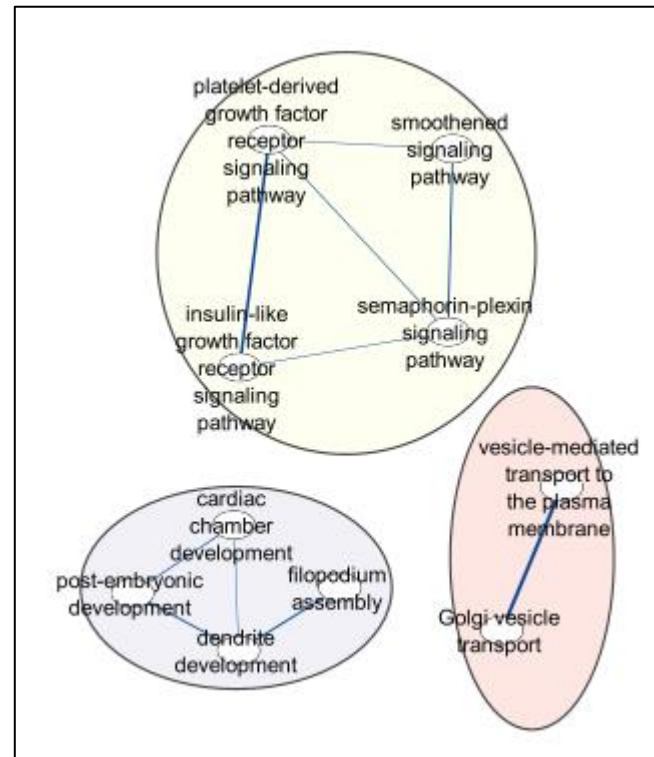

**Elements unique to HepaRG**

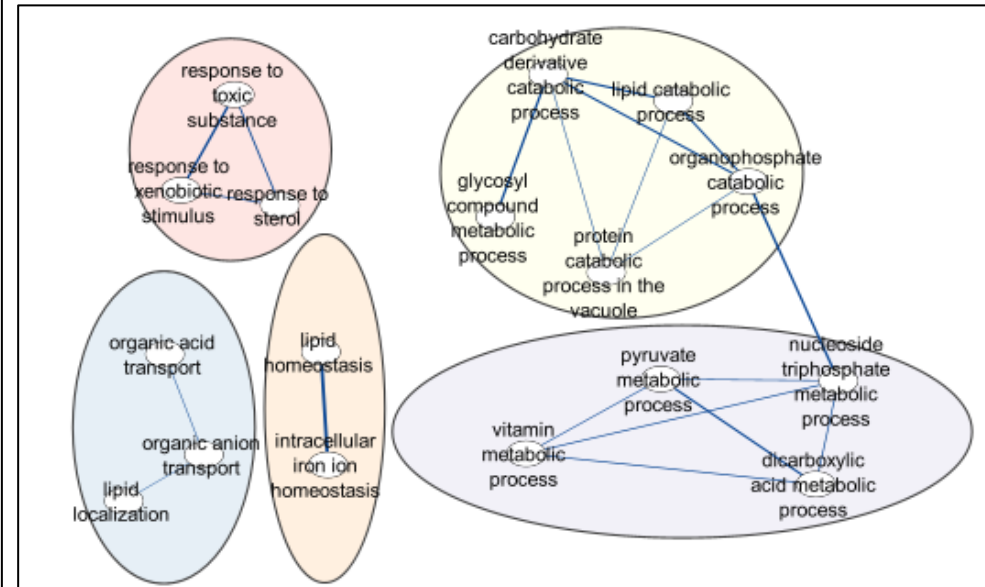

**Elements unique to primary hepatocytes**

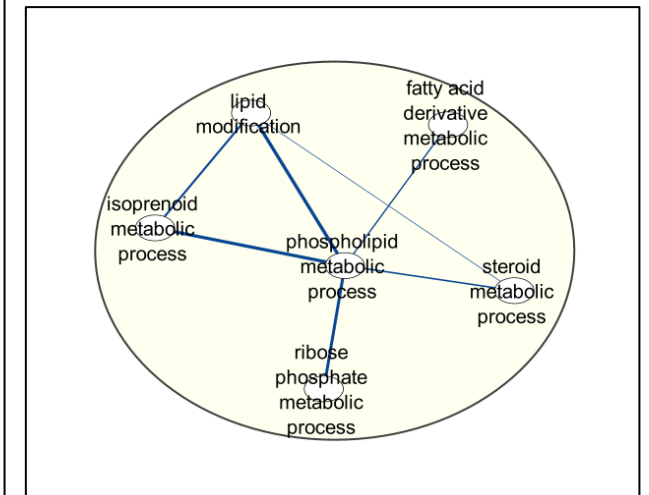

**Shared ontologies**

Supplement: S5 Fig — A, Venn diagram of nominally impacted Transcript IDs (mapped to Entrez Genes) in HepaRG cells and hepatocytes. B, Venn diagram of FDR significant Gene Ontology terms identified by GSEA in TRIBAL-suppressed HepaRG cells and hepatocytes. HepaRG (magenta) and hepatocytes (blue). C, the ontologies from B were clustered with Revigo and exported into Cytoscape for visualization. Singletons were removed to aid visualization. A complete list is shown in S1-S2 Tables. (PDF) [file pone.0322975.s007.pdf]

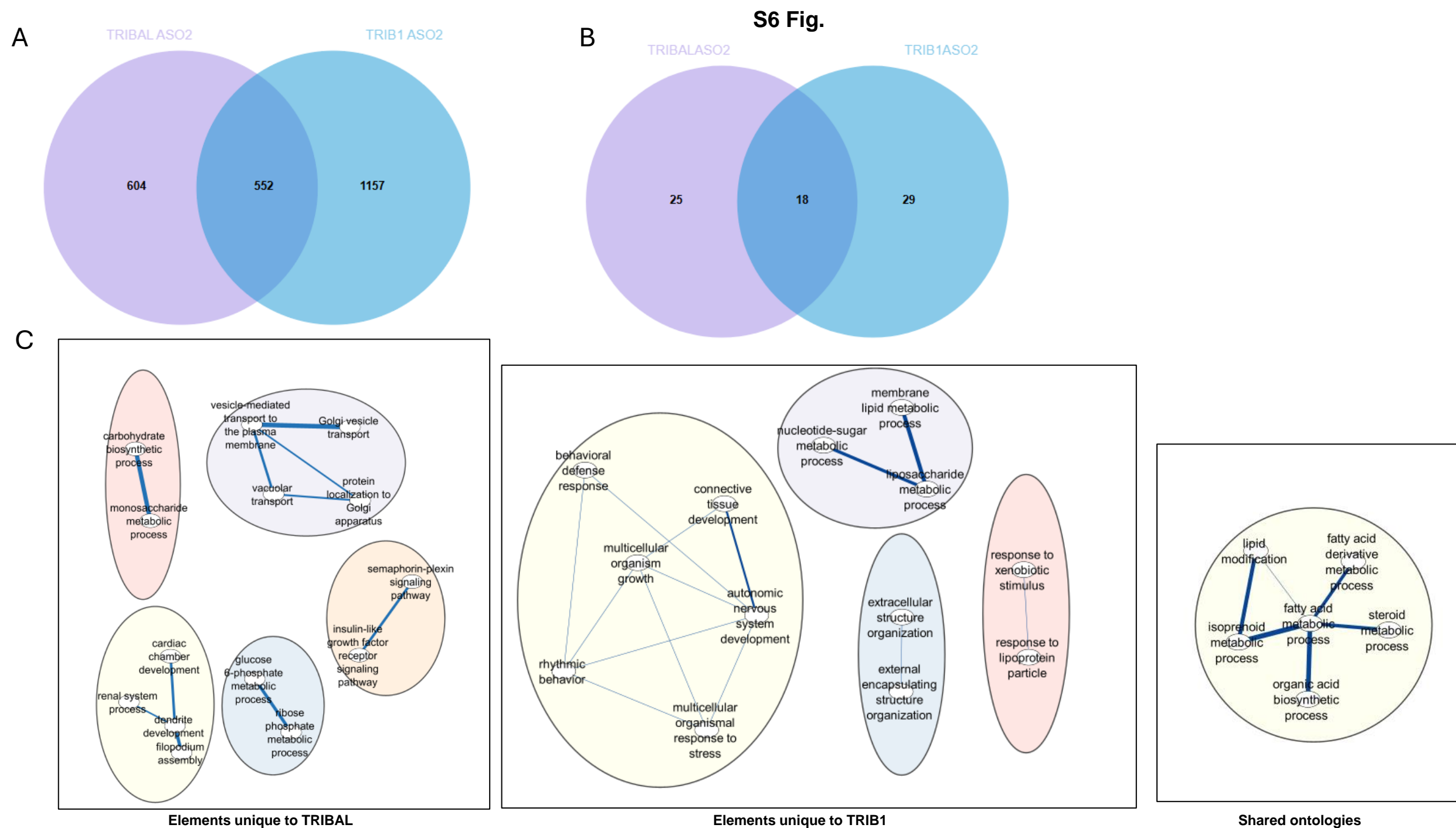

Supplement: S6 Fig — A, Venn diagram of nominally impacted Transcript IDs (mapped to Entrez Genes) in HepaRG cells targeted with TRIBAL or TRIB1 ASO2. B, Venn diagram of FDR-significant Gene Ontology terms identified by GSEA in HepaRG cells targeted with TRIBAL or TRIB1 ASO2. TRIBAL (magenta) and TRIB1 (blue). C, categories from B were clustered with Revigo and exported into Cytoscape for visualization. Singletons were removed to aid visualization. See Tables S2-S3 for the complete list of ontologies. (PDF) [file pone.0322975.s008.pdf]

S7 Fig.

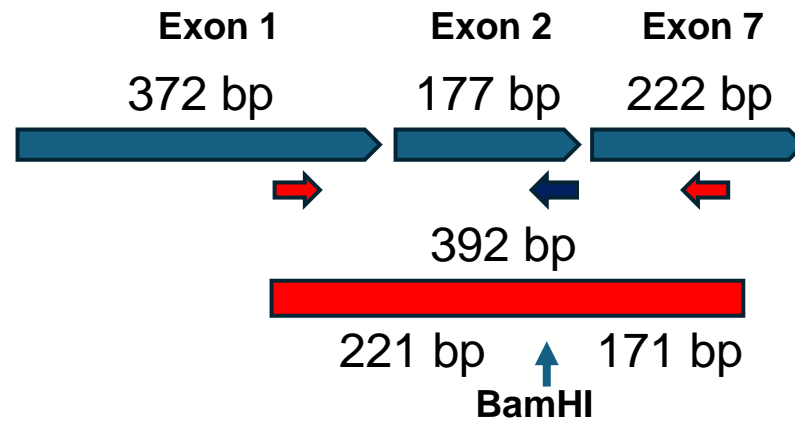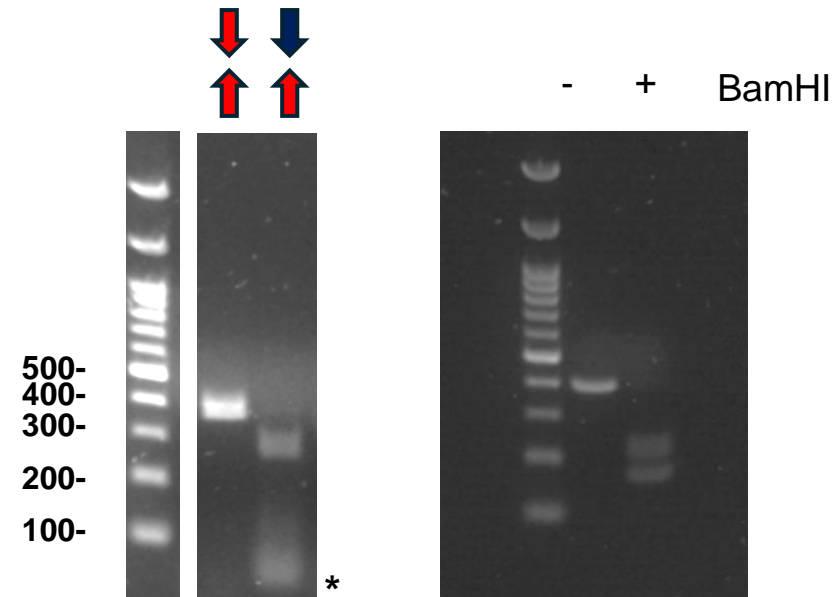

Supplement: S7 Fig — TRIBAL was amplified from cDNA derived from naïve, differentiated, HepaRG cells using PCR primers complementary to exon 1 and either exon 7 or Exon 2 (positive control) of TRIBAL. A BamHI digest was used to validate the PCR product as TRIBAL1, consisting of Exon 1, 2, and 7 of TRIBAL. Top, schematic of the PCR product and location of the BamHI site. Bottom, Agarose gels (1.5%) of the PCR products (left) and BamHI digest of the ~ 400 bp PCR product (right). * indicates primer dimers. The experiment was performed on 2 biological replicates with identical results. (PDF) [file pone.0322975.s009.pdf]

S8 Fig.

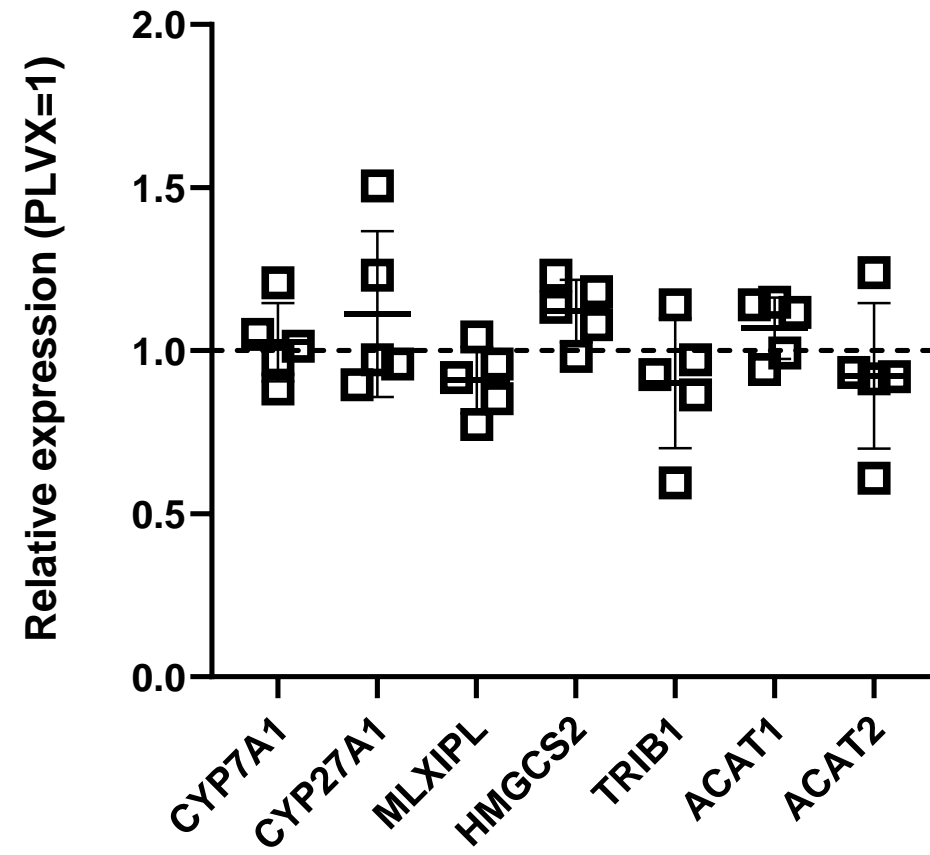

Supplement: S8 Fig — HepaRG cells were transduced with PLVXTRIBAL1 or PLVX for 72 h. RNA was then isolated and quantified by qRT-PCR. TRIBAL was upregulated by 6700 ± 4600 (S.D.). Values were internally normalized to PPIA and are expressed relative to the values from the PLVX transduced controls. Differences were not statistically different from the PLVX values (one-sample t-test using a theoretical control value of 1), except for TRIBAL (p = 0.032). (PDF) [file pone.0322975.s010.pdf]

S9 Fig.

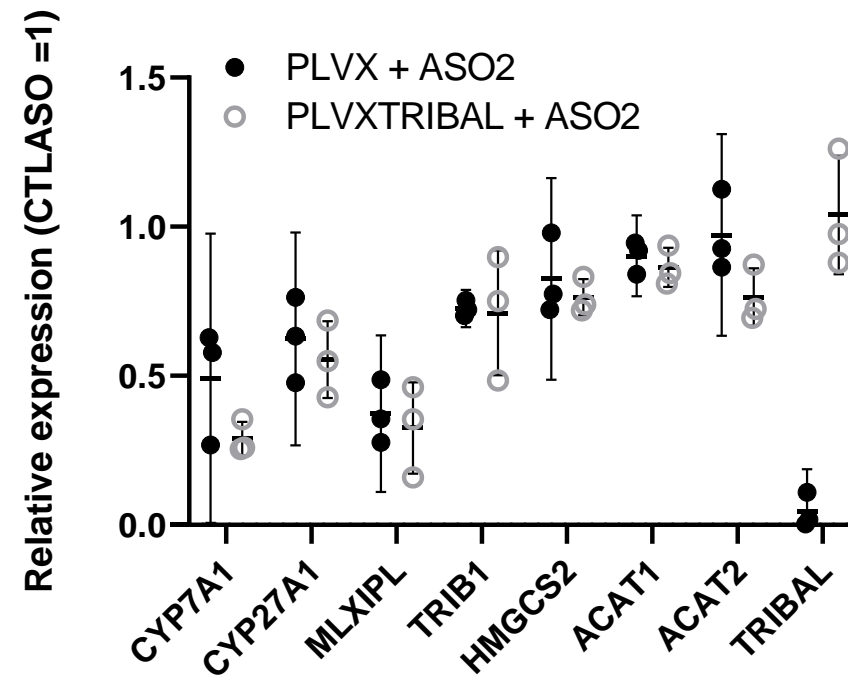

Supplement: S9 Fig — HepaRG cells were transduced with PLVXTRIBAL1 or PLVX for 48 h before treatment with TRIBAL ASO2 or CTLASO for 72 h. RNA was then isolated, converted to cDNA, and analyzed for the indicated targets by qRT-PCR. TRIBAL transduction resulted in a 460-fold (± 350, S.D.) increase in TRIBAL abundance. Each point is a biological replicate. (PDF) [file pone.0322975.s011.pdf]

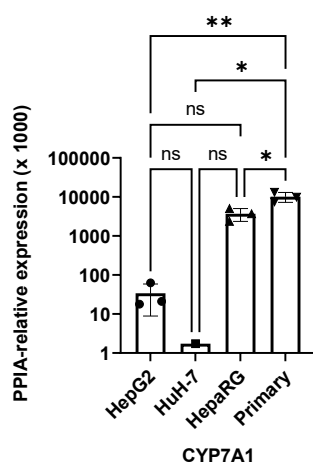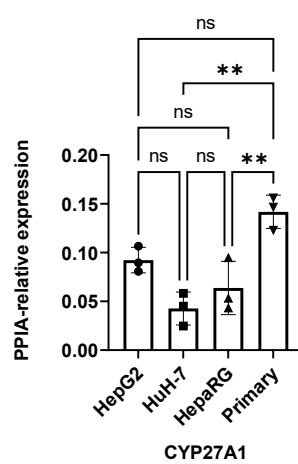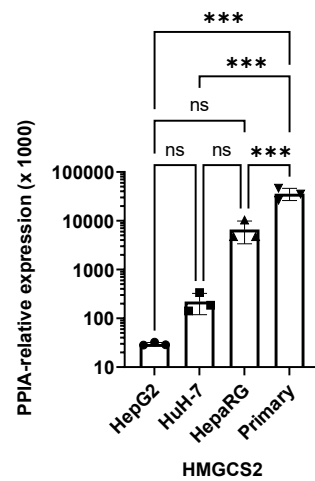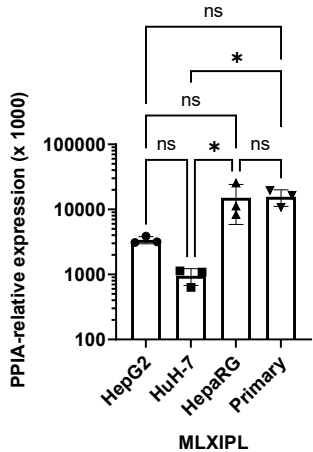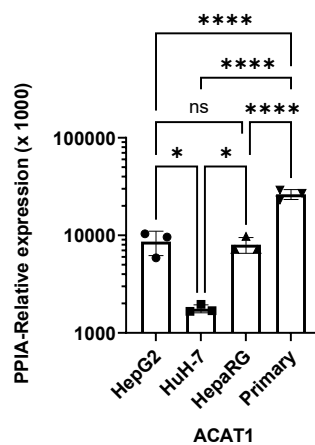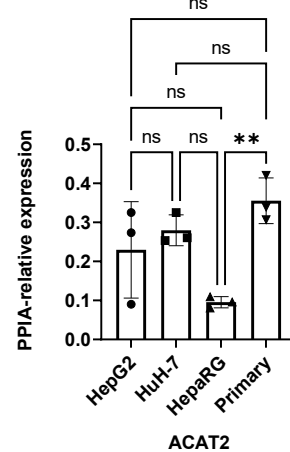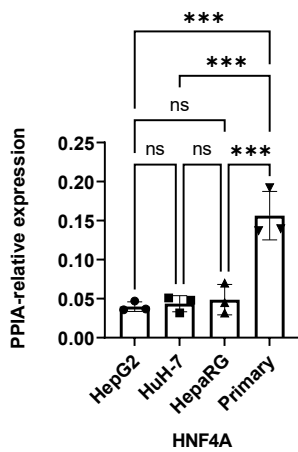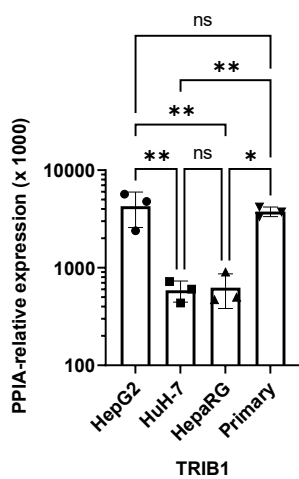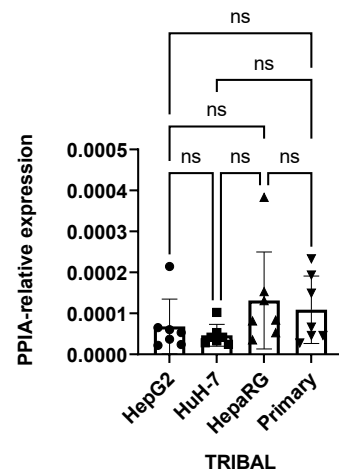

Supplement: S10 Fig — Transcript levels assessed in CTLASO-treated HepG2, HuH-7, HepaRG, and primary hepatocytes. Statistical significance was tested using one-way ANOVA, followed by a post-hoc Tukey’s multiple comparisons test. *, p < 0.05; **, p < 0.01; ***, p < 0.001, ****, p < 0.0001. (PDF) [file pone.0322975.s012.pdf]
