# Supplementary material for: Deciphering the role of the lncRNA TRIBAL in hepatocyte models
Source: PLoS One. 2025 Sep 2;20(9):e0322975. doi: 10.1371/journal.pone.0322975 (PMC12404505; doi:10.1371/journal.pone.0322975)
Supplement: S2 File — (DOCX) [file pone.0322975.s002.docx]

Antisense oligonucleotides

TRIBAL ASO (nucleotide sequence 5’ to 3’) 2’-O-(2-methoxy) ethyl-modified oligonucleotides (10 nucleotides; 5 on either side) with a phosphorothioate backbone. Obtained from Integrated DNA Technologies (IDT).

CTLASO (non-target control)

CCTTCCCTGAAGGTTCCTCC

TRIBAL ASOs:

ASO1 (targets exon 1)

CCCTTGTCATGTGGGTATTA

ASO2 (targets intron 2)

GGTGTGCAGGGTACCCTCTC

ASO5 (targets intron 2)

GGATTCTGCAGGAATTGTGG

ASO6 (targets intron 2)

TGGGAAACGGAAGGTGCGAG

ASO9 (targets intron 1)

AGGCCGTGGTCTCAGAAGCA

ASO10 (targets intron 1)

GTCCTTCAGCTCTAAAGAGG

TRIB1ASOs

ASO1 (targets exon 3)

TGCTCAGGAATGCAGAACTG

ASO2 (targets intron 2)

GTGTAGTATACATGTGCTAA

Western blotting

MLXIPL (ChREBP): Antibody #58069 (Cell Signaling Technology)

HNF4A: Rabbit mAb #3113 (Cell Signaling Technology)

TUBB: GTX11307 (GeneTex)

qPCR primers (nucleotide sequence 5’ to 3’; sense, reverse)

ACAT1

GGAGGCTGGTGCAGGAAATA

TGCCTTTTCAATGGCTCCCT

ACAT2

GCGGACCATCATAGGTTCCTT

TAGGCCTGACCCACAGATCA

CYP7A1

AAGCAAACACCATTCCAGCG

GAACCGTCCTCAAGGTGCAA

CYP27A1

AGCGATACCTGGATGGTTGG

CTTTGAGCAACGGCATGTGG

HMGCS2

CAGCAAGTTTCTTTTCATTTCG

TGGTAGAATTGCTCTCTTTGGTT

MLXIPL

CAGGGTCCAGGTTTCACACT

GAGGGAACCTCCTTTTCTGC

HNF4A

GCGGAAGAACCACATGTACTC

GGCTGCTGTCCTCATAGCTT

PPIA

ACCGTGTTCTTCGACATTGC

TTCTGTGAAAGCAGGAACCC

TRIB1

TTCAAGCAGATTGTCTCCG

CATCCACACTGGACGCGAG

TRIBAL

ACCTCCCCATGATCCAA

CATTCTGGAAGCTCTGTTGAC

Genomic PCR primers:

JUND

TCACAGTTCCTCTACCCCAA

TAGCTGCTCAGGTTCGCGTA

TRIBAL

GGCTCCATTATTCCCTCAGGCATG

AGTGAATGCTGACTTCAGGGAGGTTG

HepaRG TRIBAL validation

ACCTCCCCATGATCCAA (sense: exon 1)

CATTCTGGAAGCTCTGTTGAC (reverse: exon 2)

CCCTTGTTCGAATGGGAGTGTATG (reverse: exon 7)

CRISPR sgRNA design

crRNA sequences

nucleotide sequence 5’ to 3’; cloned 3’ of U6 promoter with BbsI (specific sequence in caps)

sg9 (recognizes coding strand)

caccAATGTCAGCTTTACGAAAGT

aaacACTTTCGTAAAGCTGACATT

sg14 (recognizes complementary strand)

caccGGGACGACAGTTCCCTCCTT

aaacAAGGAGGGAACTGTCGTCCC

TRIBAL1 sequence (cloned in PLVX)

AAAGCTGACATTTCTCATGAATAGCGAATTCCTGAGTGTGGGCAGTAGACCAAGGCCAGGAATTCTCATTAGCTGGTGATAATCAGGCATGACTTGGACTGTGGAAGGCAGCTGGGCGGAGAGGTCATAGGTATGGGCAGGGGTGAGCCACAGCTGATAGCTGGGCGCGATCTGCCTGGATTGTTACTGAAATACTAACATGCTCTTTATGGGTTAGTAACTCCCTTCACCTCCCCAACGGCTCCTACCCGCTGGGACCTTCACCTCCCCATGATCCAACAGTGAAAGATTTAATACCCACATGACAAGGGCCTTCCAGACATGGTCCAGCTGGGGTCTGATGCTGATTTGGATAGATCCATGGCAAACTGATGAAGAAATTTTGGTCAGAGAGATGAAGTAGCTCTGCCAGTACACAGCATGGAATTGAAAGCAATGGGCCTGGCACTGACTGAGAGGTGCTCACAGCTTGGGGCAGACTCCCATTTGGAGATGGGATCCTGAAGAAGCCTGGATGTTTGTGACGGCGGTCAACAGAGCTTCCAGAATGTTGTATACGAGGAACTGAGGAAAGACTACTGGGTGAGTCCATGGACCTGGCTGTGACCTGGACTGGAGTCTGGACTCCATTTGTTACCTGGCCTCCATACACTCCCATTCGAACAAGGGCCAAAATAATATTTTGGATCTCCATGTATCAACTCAGACCATGTATCCACAGTCCTTAACATTTCTGACTATGAACAGAATAAAAGCTGGGAGATACTTTGAG
