# Supplementary material for: Deciphering the role of the lncRNA TRIBAL in hepatocyte models
Source: PLoS One. 2025 Sep 2;20(9):e0322975. doi: 10.1371/journal.pone.0322975 (PMC12404505; doi:10.1371/journal.pone.0322975)
Supplement: Attachment — Submitted filename: Response to Reviewers_May 23.docx [file pone.0322975.s020.docx]

Review Comments to the Author

Please use the space provided to explain your answers to the questions above. You may also include additional comments for the author, including concerns about dual publication, research ethics, or publication ethics. (Please upload your review as an attachment if it exceeds 20,000 characters)

Reviewer #1: In this study, Sébastien and colleagues used an unconventional system to investigate the role of the lncRNA TRIBAL in hepatocyte function. However, I was unable to locate the main figures throughout the entire manuscript provided, which makes it difficult to conduct a thorough review. I would appreciate clarification to ensure there were no submission errors. Below are a few general comments that may help improve the manuscript upon resubmission or revision:

*We apologize for this oversight. Figures were indeed inexplicably omitted during submission (rather the suppl. Figures seemed to have been submitted twice). This has been corrected. As a result, clarity and readability will no doubt be improved. Again, our apologies.*

1. Terms such as “ASO” should be written in full upon first mention. Several other abbreviations also appear without prior definition and should be clarified for reader comprehension.

*Abbreviations should be defined on first appearance.*

The manuscript does not introduce or describe the differences between ASO1 and ASO2. It is unclear how the authors interpret distinct phenotypes arising from the use of these two distinct ASOs.

*The ASOs were introduced in our previous publication on TRIBAL, wherein we described their overall convergence but some apparent differences. A reference has been added (line 238) and the differences between the ASOs described in the Methods section (Under “Antisense Oligonucleotides”) and in the Discussion section.*

2. The overall readability of the manuscript is low. I strongly recommend that the authors seek input from a professional editor or experienced colleague to improve the clarity and flow of the writing. Several expressions are vague, making it difficult to follow the intended conclusions.

*Possibly vague expressions were identified and corrected. With the essential inclusion of figures, clarity and flow should be markedly improved.*

3. The figures and their legends require substantial improvement. For instance, terms such as “VP160/CG4” and “VP160/CG5” appear in the figures without any explanation in the legends and the content. I suggest the authors have project-unrelated colleagues review the figures and manuscript to ensure all components and conclusions are clearly understandable prior to resubmission.

*Thank you for your suggestion. Figure legends are now expanded to include more information.*

Reviewer #2: This article the function of lncRNA TRIBAL in the hepatocyte models, with focus on its regulation lo liver-specific transcriptional programs and then compare its function to TRIB1. Overall the experiments are designed well and excuted in a proper way. I recommend acceptance after minor revision to improve scientific clarity and completeness.

1. In the main text, the authors state that "TRIBAL1 did not protect against ASO2" and that "these experiments suggested that overexpressed TRIBAL1 was dysfunctional." (Row 330). Since this observation is an important part of the study, it would be better to briefly confirm whether the TRIBAL transcript endogenously expressed in HepaRG cells and primary hepatocytes matches the TRIBAL1 sequence used for overexpression. This could be achieved by qPCR spanning exon junctions, or by referencing existing public database annotations.

*The TRIBAL transcript used corresponded to the only form (TRIBAL1) that we previously identified in primary hepatocytes by RACE experiments. This point is now made explicit in the Results section (line 408) as well. As suggested, we have performed PCR validation in HepaRG cells using oligonucleotides targeting the first and last exon of TRIBAL1 form and demonstrate that a single band was obtained, corresponding to the predicted size of TRIBAL1. Its identity was further confirmed by restriction digest. These results indicate that TRIBAL1 is indeed the primary form in HepaRG cells. These new results have been added as S7 Fig and mentioned in the Discussion (line 519)*

2.The authors propose that differences in RNA structure might explain the inability of recombinant TRIBAL1 to mimic native TRIBAL function, based on RNAse R resistance assays (Strikingly, recombinant TRIBAL1 was highly sensitive to RNAseR, on par with the PPIA mRNA. By contrast, native TRIBAL, like U1, a highly structured snRNA, showed a near-complete resistance to RNAseR, Row 336-338). While this is a reasonable and interesting hypothesis, it remains speculative without direct structure mapping. It would strengthen the scientific rigor of the Discussion if the authors explicitly acknowledge this and suggest that future studies using RNA structure probing methods (such as SHAPE Selection to probe 2nd structure) could further confirm structural differences.

*We agree with the reviewer. This point is further raised in the Discussion, as suggested.*

While the manuscript addresses a relatively focused question, it does so with careful methodology and clear data presentation. The study will be a useful resource for researchers investigating lncRNA function and hepatocyte models.

Reviewer #3: Soubeyrand et al. present a follow-up to their previous work on long non-coding RNA TRIBAL/TRIB1AL. The authors successfully identify HepaRG cells as a suitable model system for mechanistic studies, addressing the limitations of previously used cell lines. They also elucidate the mechanism by which TRIBAL regulates key hepatocyte genes involved in metabolic function. I would like to offer the following constructive feedback:

Major points for consideration:
1. The manuscript format needs adjustment - the main figures do not appear to be integrated with the text (only supplementary figures are visible). This formatting issue makes it challenging to connect specific statements to their corresponding data, affecting the manuscript's clarity and readability.

*As mentioned above (Reviewer 1), figures were inadvertently omitted. We apologize for this oversight. Inclusion of the figures should significantly improve the clarity and readability of the manuscript.*

2. The study would benefit from additional experiments to further strengthen its novelty and impact.

*We have included additional data in response to the reviewer (Volcano plot and TRIBAL characterization in HepaRG cells). As for additional replication of presented data, see point 2 below.*

Minor points for consideration:
1. The introduction would be strengthened by several improvements. For instance, citing more recent literature on lncRNA functionality would provide a more current context than the 10-year-old Guttman et al. study. Quantitative statements with specific statistics would be preferable to qualitative terms like "likely." Additionally, reorganizing the introduction to provide general background on lncRNAs before focusing on TRIBAL would improve the logical flow.

*Thank you for this suggestion. The introduction has been expanded and now includes additional and more up-to-date references.*

2. Some statistical analyses may need additional clarification. For example, in Figure S1, certain groups appear to have only 3 samples, which may impact statistical robustness.

*Most of our experiments were performed 3 or 4 times by design, as we were expecting to observe large effect sizes, as we previously observed with primary hepatocytes. We have not attempted to add additional rounds of experiments, as these experiments were consistent with a null hypothesis. However, we readily acknowledge that with a nominal number of repeats, power is low and that subtle but genuine differences (albeit of uncertain biological significance) could be missed.*

3. The conclusion stating that "TRIBAL suppression in HepaRG resulted in ~5 times fewer nominally significant hits than in hepatocytes, suggesting either the possible loss of TRIBAL targets in HepaRG or lower quality data" could be strengthened. If data quality is a concern, the rationale for using this dataset/model should be further justified.

*After re-examining our data (more specifically the volcano plots), it became apparent that the main difference between HepaRG and Hepatocytes was related to the fold-change, not the p-values (i.e. changes in HepaRG were of much lower magnitude). Thus, we are reasonably confident that lower quality data is not an issue but that HepaRGs were less impacted. This sentence has been modified and the volcano plots are now included (as S4. Fig; Fig S4 has been moved to S5 etc.)*

4. For the GO analysis, could you please provide additional details regarding p-values, the number of genes in each set, and any analysis of gene overlap between categories?
The observation that TRIBAL transduction did not restore function in TRIBAL-suppressed cells warrants further Discussion.

*The p-values are available in the Supplemental Tables. P=values were obtained, as is typical for GSEA, by permutation-based analysis of ranked lists. The p-value was calculated by determining how frequently the ES from the actual ranking is greater than that for the random permutations. WebGestalt uses a typical 1000 permutations to calculate the significance of the enrichment score (ES). This has been included in the Methods section. The tables were expanded to include the number of genes in each set (rather than just the leading edge transcripts). Although the gene overlap between categories is not shown (given the large number of categories), the ENTREZ gene IDs populating these categories are now included in the tables.*

5. Could the authors elaborate on whether this affects TRIBAL's potential as a therapeutic target? Additionally, addressing the structural differences and their potential causes would be valuable.

*We do not believe that TRIBAL targeting is a viable therapeutic approach, on both theoretical and practical grounds. For one, as TRIBAL is important to sustain normal liver function, TRIBAL suppression is predicted to be toxic. As for its overexpression, for example as a treatment for steatotic liver disease, our data indicate that this is not feasible since transduced TRIBAL1 cannot impact the targets of TRIBAL. Assuming that transcriptional activation approaches may be optimized to activate the endogenous locus, we note that genetic predisposition to higher TRIBAL abundance associate with increased cardiovascular risk, suggesting that higher TRIBAL expression may be detrimental. On practical grounds, a major limitation resides in the paucity of actionable animal models, as TRIBAL is only expressed in primates. Although one could envision hybrid cancer models (e.g. human tumors in mouse models), the lack of in vitro impact of the ASOs on HuH-7, HepG2, and undifferentiated HepaRGs suggests that (liver) cancers may not be responsive to TRIBAL targeting.*

*We have expanded our Discussion (last paragraph) to suggest dedicated approaches to test structural differences between the transduced and endogenous forms. Although we propose models to account for these differences, they remain speculative until structural differences are confirmed.*
