# Supplementary material for: Deciphering the role of the lncRNA TRIBAL in hepatocyte models
Source: PLoS One. 2025 Sep 2;20(9):e0322975. doi: 10.1371/journal.pone.0322975 (PMC12404505; doi:10.1371/journal.pone.0322975)
Supplement: Attachment — Submitted filename: Rebuttal_July 17.docx [file pone.0322975.s021.docx]

Reviewer #1: This manuscript by Soubeyrand et al. aims to elucidate the role of the TRIBAL in hepatocyte models, focusing on its regulatory relationship with TRIB1 and its impact on liver-specific transcriptional programs. While the study presents a substantial amount of data, however, I find several issues that limit the current conclusions and overall impact of this work.

**Thank you for a very detailed review. We have addressed specific concerns as detailed below and have rewritten several parts of the manuscript to improve clarity and readability.**

Major Points:
1. If the TOIs were selected based on previous publications, please cite the relevant references. Additionally, the title for the first paragraph of the result seems inappropriate—I did not find any content specifically addressing the optimal growth of HepG2 or HuH-7 cells. Although the authors mention, in the Methods section that they have explained the differences between ASO1 and ASO2 (and additional ASOs in figure 4), it remains unclear what drives the phenotypic differences. Are ASO1 and ASO2 targeting different functional domains, leading to distinct biological effects? Or is the difference purely due to targeting efficiency? Please summarize this in a concise sentence to help readers understand the nature of these ASOs.

**The relevant references have been added. Regarding optimal growth, we aimed to convey the meaning of optimal under normal growth conditions (standard media), rather than the optimal growth condition itself. For clarity, ‘optimal’ has been removed.**

**We have expanded the Methods section (Antisense oligonucleotides) to include a more complete description of the ASOs. As explained in that section, ASO1 targets exon 1 of TRIBAL, whereas all the others target either of 2 introns. We did find that ASO1 was modestly less effective at reducing TRIBAL levels, but significantly less effective at suppressing the TOIs. We previously demonstrated (as mentioned in the Discussion lines 496-512) that an additional ASO targeting exon 1 (not used in this work) was shown to have a comparable impact as ASO2 but resulted, on average, in a more modest ASO1-like impact in primary hepatocytes. Thus, we hypothesize that intron targeting has additional effects related to targeting of the nascent transcript, as noted in the Discussion.**

2. What are the baseline expression levels of the TOIs in each cell line used in the study (HepG2, Huh-7 vs HepaRG)? Are there inherent expression differences without TRIBAL manipulation?

**An important point. We have added a direct comparison amongst all the TOIs in HepG2, HuH-7, HepaRG and primary hepatocytes, obtained in CTLASO-treated cells. Note that these values were normalized to PPIA expression (which we have used as a robust housekeeper in our work over the years and is unaffected by TRIBAL suppression based on transcription array evidence). The additional figure (S10 Fig) was integrated intp the Discussion (line 485), where it seemed to fit best.**

3. ASO9 appears to exhibit stronger suppression than ASO2, outperforming it in some assays. Therefore, ASO9 should be included as a repeat along with ASO2 in subsequent experiments to validate findings, particularly when testing TRIBAL1 functionality.

**ASO9 may have a greater impact than ASO2 (Figure 4) on some transcripts (CYP7A1, HGMGCS2, ACAT1), but the difference is small (median 1.1-fold, average 1.2-fold). Thus, given the already sizeable impact of ASO2 and the lack of rescue with TRIBAL1 OE, we believe that repeated experiments with ASO9 are unlikely to inform on the main point, i.e. the incapacity of TRIBAL1 to rescue the impact of ASO-mediated TRIBAL knockdown.**

4. Comparing Figures 2, 4, and 5 reveals inconsistencies in the effects of ASO2 on TRIB1 expression. In Figures 2 and 5, ASO2 does not significantly affect TRIB1 expression in HepaRG cells, whereas in Figure 4, the effect appears significant. Moreover, published data using primary hepatocytes also show no significant reduction in TRIB1 with ASO2 treatment. These discrepancies call into question the conclusion presented in lines 288–291. Additionally, in Figure 5B, suppression of TRIB1 using either ASO1 or ASO2 does not alter TRIBAL expression. Do you believe the regulation of TOIs by TRIBAL suppression is independent or dependent on TRIB1? Please clarify.

**Our data demonstrates that TRIB1 expression was overall reduced, albeit not always enough to achieve statistical significance in any single round of 3-4 experiments. In Figure 2, an approximately 25% reduction, but not significant (1 out of 4 may be an outlier). In Figure 5 (hepatocytes), the reduction is also approximately 20%, and in Figure 4, the impact is more pronounced, on average, at 40%. Thus, on aggregate, TRIBAL suppression correlates with reduced TRIB1 expression.**

**However, we believe that the regulation of the TOIs by TRIB1 and TRIBAL occurs largely independently, based on ASO evidence showing that (as rightly pointed out), TRIBAL suppression can impact TOI, even when TRIB1 expression is modestly (or not) impacted (Fig 5A, TRIBAL ASO2). Similarly, TRIB1 suppression impacts the TOI even with minimal changes to TRIBAL. However, this apparent independence of function should not be misconstrued to imply that they act on different targets. This point has been clarified in the first two paragraphs of the Discussion.**

5. In Figure 6, a correlation between TRIBAL and TRIB1 expression does not imply co-regulation. The conclusion that TRIBAL regulates TRIB1 based on expression correlation is speculative and not mechanistically justified.

**We agree that a conclusion to the effect that TRIBAL can regulate TRIB1 was probably too speculative and have revised the text accordingly. The correlation is consistent (but not necessarily causative) with both transcripts sharing similar regulators (what we intended to convey with the phrase “co-regulation”). The Results section has been rewritten accordingly (lines 337-339).**

Minor points:
1. Some figures can be combined to improve reading continuity—for example, Figures 2 and 3 could be merged.

**Thank you for your suggestion. However, we feel that keeping RNA and protein results separately reduces confusion. The flow should improve significantly once the manuscript reaches the galley proof stage and the text-embedded legends are removed.**

2. Figure legends and tables are currently embedded within the main text; they should be formatted appropriately according to journal guidelines.

**Our understanding is that, according to the PLOSONE journal guidelines, tables and legends should be integrated within the submitted manuscript’s main text.**
